# B.1.351 SARS-CoV-2 Variant Exhibits Higher Virulence but Less Viral Shedding than That of the Ancestral Strain in Young Nonhuman Primates

**DOI:** 10.1128/spectrum.02263-22

**Published:** 2022-09-07

**Authors:** Yu Bai, Qian He, Jinghuan Yang, Shuaiyao Lu, Qunying Mao, Fan Gao, Lianlian Bian, Jialu Zhang, Chaoqiang An, Jianyang Liu, Xing Wu, Wenhai Yu, Zhongfang Wang, Xiaozhong Peng, Junzhi Wang, Zhenglun Liang, Miao Xu

**Affiliations:** a Division of Hepatitis and Enterovirus Vaccines, Institute of Biological Products, National Institutes for Food and Drug Control, NHC Key Laboratory of Research on Quality and Standardization of Biotech Products, NMPA Key Laboratory for Quality Research and Evaluation of Biological Products, Beijing, People’s Republic of China; b Institute of Medical Biology, Chinese Academy of Medicine Sciences & Peking Union Medical College, Kunming People’s Republic of China; c Guangzhou Laboratory, Guangzhou, China; Shandong First Medical University

**Keywords:** SARS-CoV-2 variant, viral distribution, virulence, viral shedding, nonhuman primate

## Abstract

We investigated the distribution, virulence, and pathogenic characteristics of mutated SARS-CoV-2 to clarify the association between virulence and the viral spreading ability of current and future circulating strains. Chinese rhesus macaques were infected with ancestral SARS-CoV-2 strain GD108 and Beta variant B.1.351 (B.1.351) and assessed for clinical signs, viral distribution, pathological changes, and pulmonary inflammation. We found that GD108 replicated more efficiently in the upper respiratory tract, whereas B.1.351 replicated more efficiently in the lower respiratory tract and lung tissue, implying a reduced viral shedding and spreading ability of B.1.351 compared with that of GD108. Importantly, B.1.351 caused more severe lung injury and dramatically elevated the level of inflammatory cytokines compared with those observed after infection with GD108. Moreover, both B.1.351 and GD108 induced spike-specific T-cell responses at an early stage of infection, with higher levels of interferon gamma (IFN-γ) and tumor necrosis factor alpha (TNF-α) in the B.1.351 group and higher levels of interleukin 17 (IL-17) in the GD108 group, indicating a divergent pattern in the T-cell-mediated inflammatory “cytokine storm.” This study provides a basis for exploring the pathogenesis of SARS-CoV-2 variants of concern (VOCs) and establishes an applicable animal model for evaluating the efficacy and safety of vaccines and drugs.

**IMPORTANCE** One of the priorities of the current SARS-CoV-2 vaccine and drug research strategy is to determine the changes in transmission ability, virulence, and pathogenic characteristics of SARS-CoV-2 variants. In addition, nonhuman primates (NHPs) are suitable animal models for the study of the pathogenic characteristics of SARS-CoV-2 and could contribute to the understanding of pathogenicity and transmission mechanisms. As SARS-CoV-2 variants continually emerge and the viral biological characteristics change frequently, the establishment of NHP infection models for different VOCs is urgently needed. In the study, the virulence and tissue distribution of B.1.351 and GD108 were comprehensively studied in NHPs. We concluded that the B.1.351 strain was more virulent but exhibited less viral shedding than the latter. This study provides a basis for determining the pathogenic characteristics of SARS-CoV-2 and establishes an applicable animal model for evaluating the efficacy and safety of vaccines and drugs.

## INTRODUCTION

The coronavirus disease 2019 (COVID-19), caused by severe acute respiratory syndrome coronavirus 2 (SARS-CoV-2), has led to a global pandemic with more than 490 million confirmed cases and more than 6 million deaths as of 8 April 2022, as reported by the World Health Organization (WHO) (1). The significant morbidity and mortality of COVID-19 pose a huge threat to human health. Moreover, the emergence of SARS-CoV-2 variants, especially the WHO-defined variants of concern (VOCs), which manage to escape immunity induced by current vaccines to various degrees, have worsened the COVID-19 epidemic situation worldwide (2).

Therefore, there is an urgent need to develop effective vaccines and drugs against SARS-CoV-2. Animal models are crucial tools for elevating the safety and efficacy of vaccines and drugs. To this end, human angiotensin-converting enzyme 2 (ACE2) transgenic mouse, hamster, ferret, and nonhuman primate (NHP) pathological models of SARS-CoV-2 infection have been successively established (3–10). Among these, NHPs are phylogenetically related to humans and share a wide range of viral pathogens, often mimicking the clinical presentation of human infections (6, 11, 12). In addition, their immune system, respiratory system anatomy, and tissue structure are very similar to those of humans (13–15). Hence, NHPs represent one of the most suitable animal models for vaccine and drug evaluation. Before the emergence of the currently widespread VOCs, most preclinical NHP models for SARS-CoV-2 infection were established using the prototype strain (14, 16); this has led to a lack of data regarding the pathogenicity of different variant strains in NHPs (17). Consecutively, assessing the effectiveness of a preventive or treatment measure against variants in animal models based on the pathogenic characteristics of the prototype strain lacks scientific validation. Therefore, it is imperative to develop and analyze a SARS-CoV-2 variant infection model using NHPs.

Importantly, one of the priorities of current epidemic prevention work is to determine the transmission ability, virulence, and pathogenic characteristics of SARS-CoV-2 variants, so as to formulate possible vaccination and disease control strategies against current and future circulating strains. However, the mechanisms by which these concerned mutations affect the pathogenic characteristics of SARS-CoV-2, including viral dissemination, virulence, and viral shedding, have not yet been determined (2, 18). Here, we developed an NHP model, which highly resembles humans in structure, function, and metabolism, to comprehensively study the pathogenicity characteristics of the SARS-CoV-2 variant B.1.351 and the ancestral strain GD108.

## RESULTS

### Clinical signs in macaques infected with B.1.351 and GD108.

**FIG 1:**
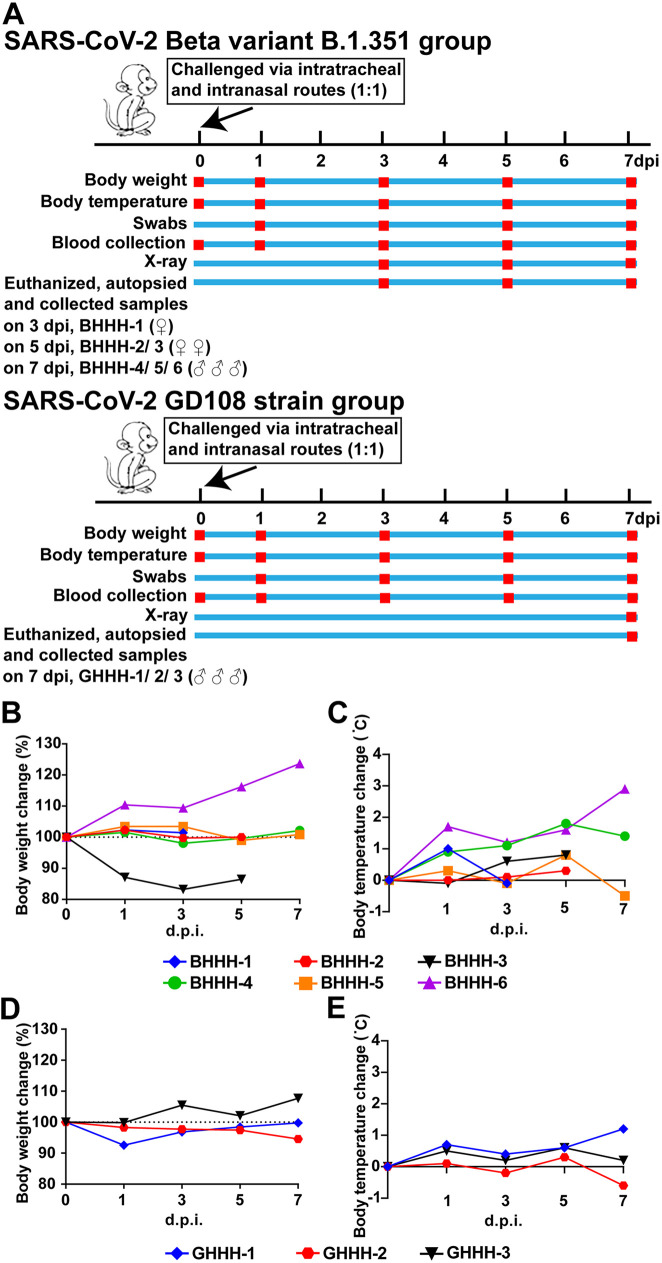
Animal experimental procedure and clinical signs of SARS-CoV-2 infection in macaques. (A) Process of animal infection with the B.1.351 and GD108 strains. (B) Changes in body weight in the B.1.351 group. (C) Changes in body temperature in the B.1.351 group. (D) Changes in body weight in the GD108 group. (E) Changes in body temperature in the GD108 group. For the B.1.351 group, *n* = 6 from 0 to 3 dpi, *n* = 5 at 5 dpi, and *n* = 3 at 7 dpi. For the GD108 group, *n* = 3 from 0 to 7 dpi.

**FIG 2:**
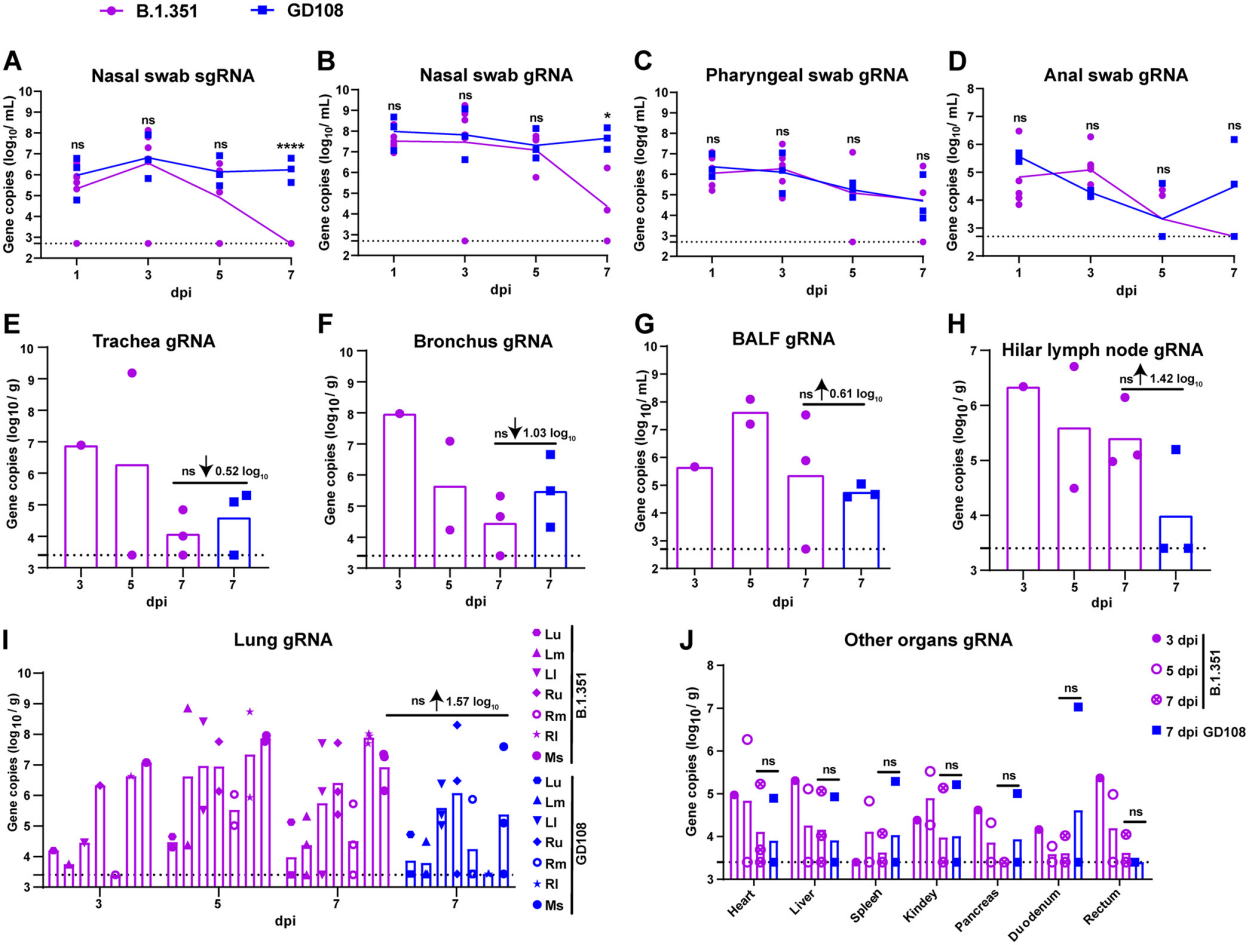
Viral gene copy numbers in macaques infected with B.1.351 and GD108. (A) sgRNA copy number in nasal swabs. For B.1.351, *n* = 6. For GD108, *n* = 3. (B to D) gRNA copy number in nasal, pharyngeal, and anal swabs. For B.1.351, *n* = 6. For GD108, *n* = 3. (B) Nasal swab; (C) pharyngeal swab; (D) anal swab. (E to J) gRNA copy number in trachea, bronchus, BAL fluid, hilar lymph nodes, lungs, and other organs. (E) Trachea; (F) bronchus; (G) BAL fluid; (H) hilar lymph nodes; (I) lungs. The statistical analysis was performed in mixed 6 lobes of lung between the B.1.351 and the GD108 groups. (J) Other organs. For B.1.351, *n* = 1 at 3 dpi, *n* = 2 at 5 dpi, and *n* = 3 at 7 dpi. For GD108, *n* = 3 at 7 dpi. Lu, left upper lobe; Lm, left middle lobe; Ll, left lower lobe; Ru, right upper lobe; Rm, right middle lobe; Rl, right lower lobe; Ms, mixed 6 lobes; ↓, decrease; ↑, increase. ns, nonsignificant; *, *P < *0.05; ****, *P < *0.0001.

We observed that in the B.1.351 group, one out of six macaques exhibited an obvious decrease in body weight, with BHHH-3 showing the most obvious drop of 16.79% (Fig. 1B). In addition, the body temperature was increased in all six macaques to various degrees (increases of 0.3 to 2.9°C) from 0 to 7 days postinfection (dpi) (Fig. 1C). In the GD108 group, we found that the body weight was decreased in one out of three macaques, with GHHH-2 showing a drop of 5.44% at 1 dpi (Fig. 1D). Moreover, the body temperature was increased in all macaques to various degrees (increases of 0.3 to 1.2°C) from 0 to 7 dpi (Fig. 2E). These findings indicate that both B.1.351 and GD108 led to a decrease in body weight and increase in body temperature of macaques to various degrees. In addition, we detected that both B.1.351 and GD108 caused hematological changes in macaques, such as in the number of immune and red blood cells, concentration of immune globulins, content of metabolic enzymes and metabolites (aspartate transaminase [ALT], blood urea nitrogen [BUN], and creatinine [CRE-E]), and activation of the complement system (see Fig. S1 and S2 at https://figshare.com/s/8204af70a23e2a7da8c8). Collectively, these findings suggest that macaques were successfully infected by B.1.351 and GD108 and presented clinical signs of acute viral infection.

### Viral distributions in macaques infected with B.1.351 and GD108.

To determine the viral distribution in macaques, we measured the level of SARS-CoV-2 subgenomic RNA (sgRNA) in nasal swabs, as well as the level of genomic RNA (gRNA) in respiratory and digestive tract swabs and that in different organs using quantitative real-time PCR (qRT-PCR). We found that the level of sgRNA in the nasal swabs from both groups reached a peak at 3 dpi and then gradually declined, approaching the lower limit of detection (LLOD) at 7 dpi and thereafter in the B.1.351 group, which was significantly lower than that in the GD108 group at 7 dpi (*P < *0.0001) (Fig. 2A). The level of gRNA in the nasal, pharyngeal, and anal swab samples showed peaks at 1 and 3 dpi in both groups and then gradually declined, approaching the LLOD at 7 dpi in the B.1.351 group, which was also lower than that in the GD108 group to various degrees; the level of gRNA in the nasal swab samples of the B.1.351 group especially was markedly lower than that of the GD108 group (*P < *0.05) (Fig. 2B through D). In addition, we noticed that the levels of gRNA in the trachea, bronchus, bronchoalveolar lavage fluid (BAL fluid), hilar lymph nodes, and lung reached peaks at 3 and 5 dpi in the B.1.351 group (Fig. 2E through I). Notably, the levels of gRNA in the trachea and bronchus of macaques in the B.1.351 group were lower than those in the GD108 group at 7 dpi to various degrees (Fig. 2E and F). On the contrary, the levels of gRNA in BAL fluid, hilar lymph nodes, and lung (the whole lung and six individual lobes) in the B.1.351 group were higher than those in the GD108 group at 7 dpi to various degrees (Fig. 2G through I). Regarding the viral distribution in other organs, we did not identify any differences between the two groups at 7 dpi (Fig. 2J). These results indicated that both B.1.351 and GD108 replicated in the respiratory system and other organs and could be excluded from the respiratory and digestive tracts. The obvious difference between the two viral strains was that the replication capacity of B.1.351 was weaker than that of GD108 in the upper respiratory tract, whereas it was stronger in the lower respiratory tract of macaques.

### B.1.351 caused more severe pathological changes in macaques than those after GD108 infection.

**FIG 3:**
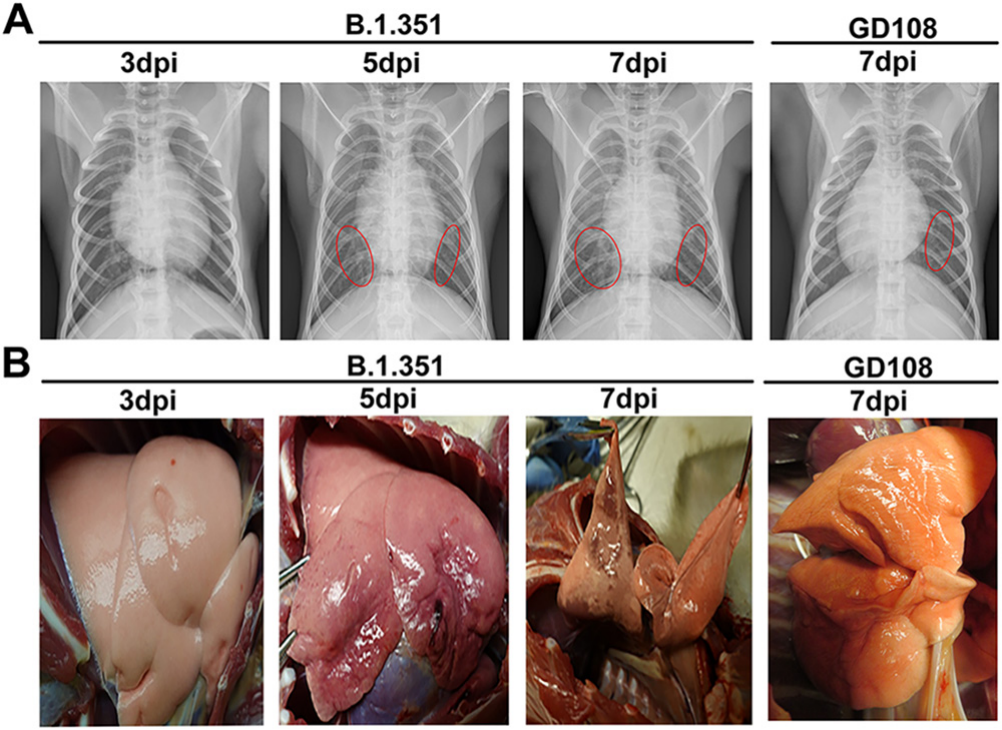
Radiological and gross pathological autopsies in macaques infected with B.1.351 and GD108. (A) Chest radiographs in macaques infected with B.1.351 from 3 to 7 dpi and GD108 at 7 dpi. (B) Gross pathological autopsies in macaques infected with B.1.351 from 3 to 7 dpi and GD108 on 7 dpi.

The primary reason for death caused by SARS-CoV-2 infection in the clinical setting is acute lung injury. Thus, we performed lung radiography and observed the pathological changes in macaque lungs postinfection. In the B.1.351 group, we observed lung abnormalities in the form of nodules, masses, and interstitial patterns at 5 and 7 dpi, as indicated by chest radiographs. Overall comparison of chest radiographs showed that the pulmonary abnormalities were more severe as the infection progressed. In the GD108 group, lung abnormalities in the form of nodules, masses, and interstitial patterns were observed in the lung postinfection. Nevertheless, degrees of abnormalities in the GD108 group were obviously slighter than those of the B.1.351 group (Fig. 3A).

In the B.1.351 group, lung gross pathological anatomy revealed severe congestion and hemorrhage and slight edema at 5 and 7 dpi; these changes were more severe on the 7th dpi than on the 5th dpi in the B.1.351 group. In the GD108 group, congestion, hemorrhage, and slight edema were observed in the lung postinfection. Nevertheless, degrees of abnormalities in the GD108 group were obviously slighter than those in the B.1.351 group (Fig. 3B). We verified that the lung abnormalities observed in chest radiographs were consistent with the results of lung gross pathological anatomy.

**FIG 4:**
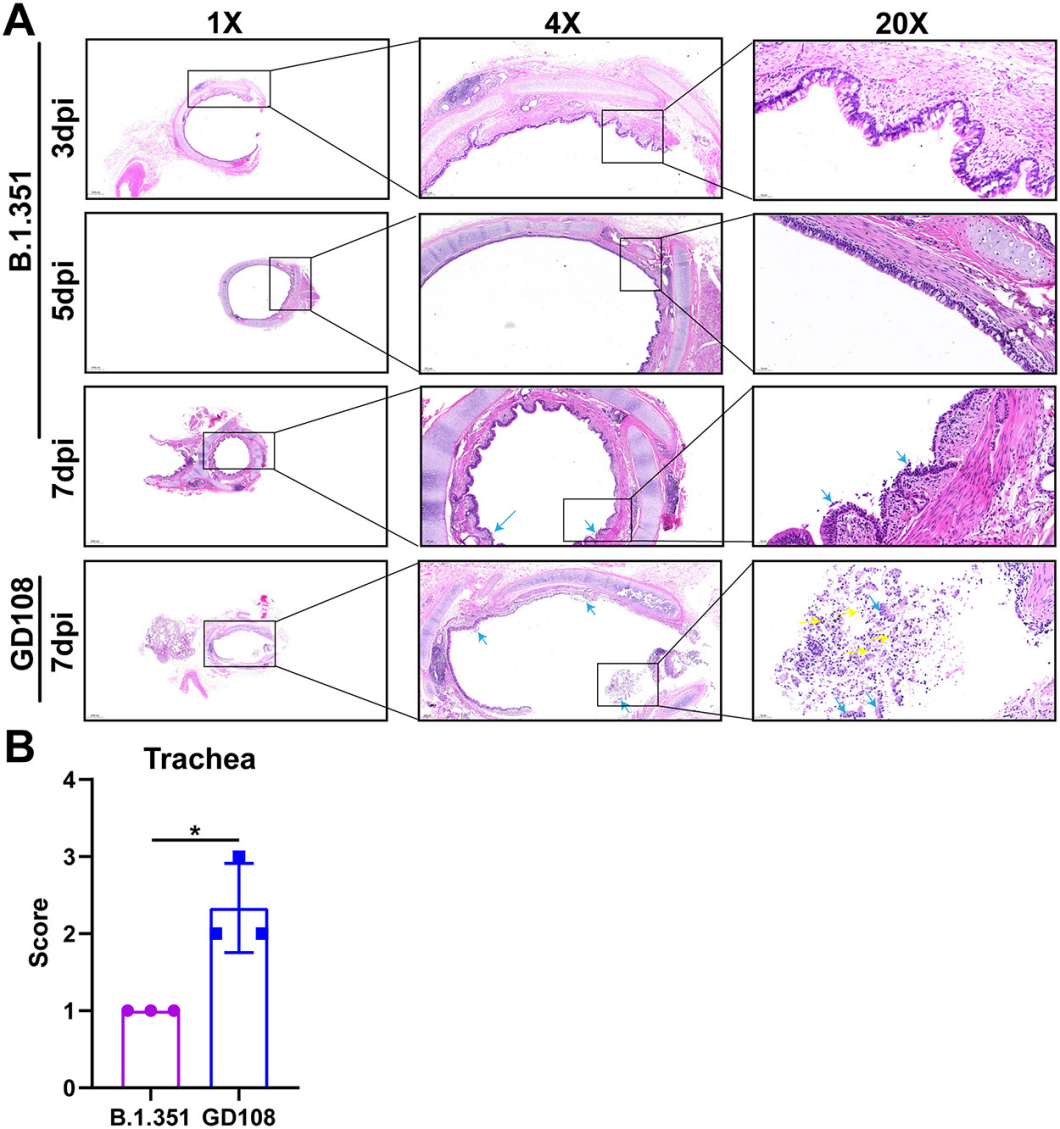
Histopathological changes of tracheas in macaques infected with B.1.351 and GD108. (A) Histopathological images of tracheas of macaques infected with B.1.351 and GD108. (B) Trachea histopathological scores in B.1.351 and GD108 groups. For B.1.351, *n* = 1 at 3 dpi, *n* = 2 at 5 dpi, and *n* = 3 on 7 dpi. For GD108, *n* = 3 at 7 dpi. Yellow arrow, inflammatory cell infiltration; light-blue arrow, epithelium shedding. *, *P* < 0.05.

**FIG 5:**
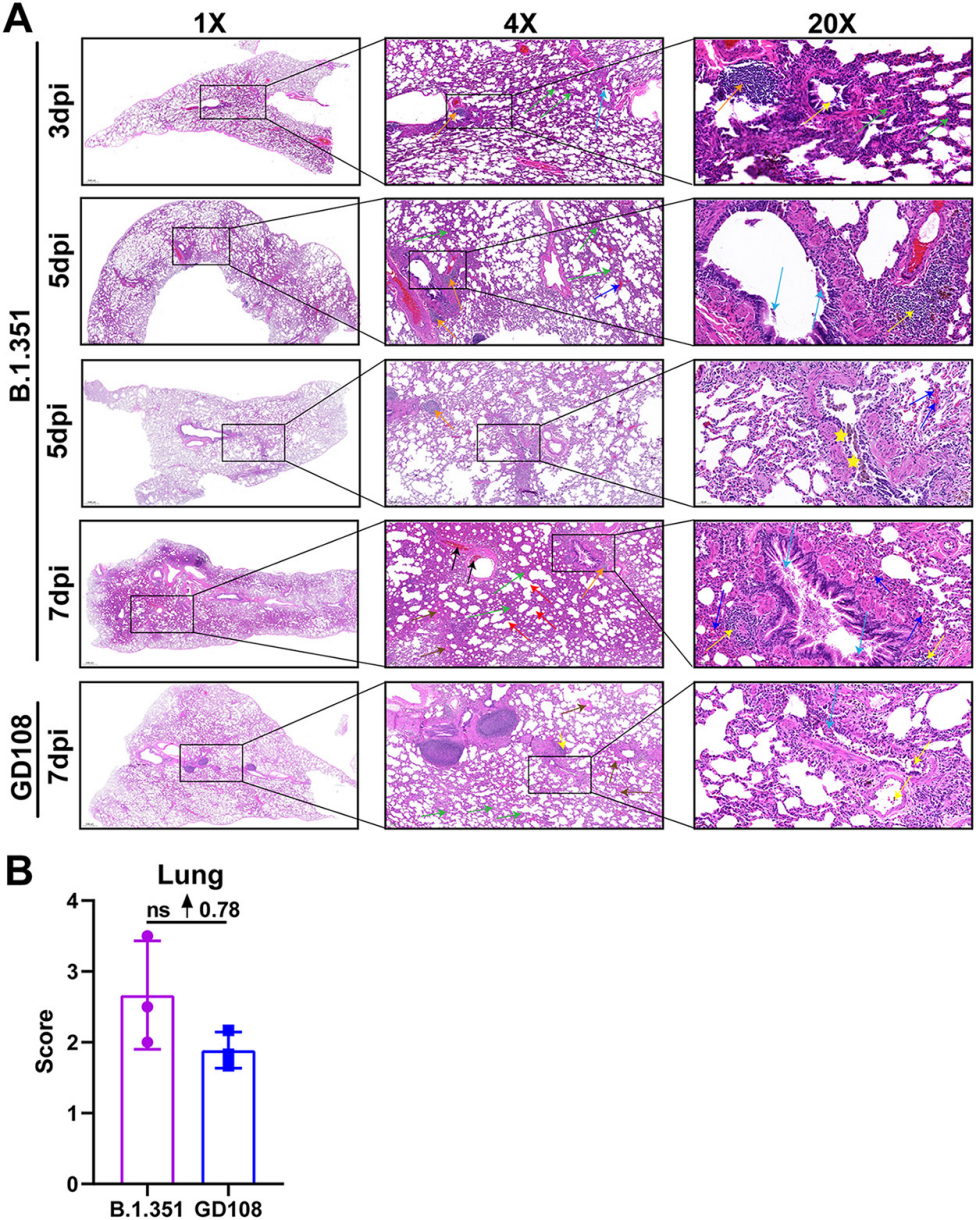
Histopathological changes of lungs in macaques infected with B.1.351 and GD108. (A) Histopathological images of lungs of macaques infected with B.1.351 and GD108. (B) Lung histopathological score in B.1.351 and GD108 groups. For B.1.351, *n* = 1 at 3 dpi, *n* = 2 at 5 dpi, and *n* = 3 at 7 dpi. For GD108, *n* = 3 at 7 dpi. Yellow arrow, inflammatory cell infiltration; light-blue arrow, epithelium shedding; black arrow, thrombus; green arrow, thickening in alveolar walls; red arrow, emphysema; orange arrow, peribranchial lymph node; yellow star, macrophages engulfing pigment particles; deep blue, hemorrhage; brown arrow, eosin-stained thick fluid; ↑, increase; ns, nonsignificant.

Next, we performed hematoxylin and eosin (H&E) staining to observe histopathological changes in the trachea and lungs of macaques postinfection. In the B.1.351 group, we did not detect any histopathological change in the trachea on the 3rd and 5th dpi, whereas we found some necrotic epithelium shedding into the bronchial lumen on the 7th dpi. In the GD108 group, we detected severe necrotic epithelium shedding and infiltration of inflammatory cells into the bronchial lumen on the 7th dpi. The trachea histopathological score of the B.1.351 group was markedly lower than that of the GD108 group (*P < *0.05) (Fig. 4A and B). Most importantly, we observed obvious histopathological changes in the lungs of macaques following infection with either B.1.351 or GD108. In particular, we noticed thickened alveolar walls, bronchial epithelium shedding, emphysema, hemorrhage into the alveolar space, and infiltration of inflammatory cells into the bronchus and alveolar space in animals in the B.1.351 group. In addition, the peribranchial lymphatic nodule was filled with abundant inflammatory cells. Notably, the alveoli were filled with eosin-stained thick fluid. We also observed macrophage phagocytosis of pigment granules and pulmonary thrombosis on the 5th and 7th days postinfection, respectively. Overall, the severity of pathological changes gradually increased with the disease course from 3 to 7 dpi. We also observed thickened alveolar walls, bronchial epithelium shedding, and infiltration of inflammatory cells into the bronchus and alveolar space in the lungs of animals in the GD108 group. Moreover, the peribranchial lymphatic nodule was filled with inflammatory cells, and the alveoli were filled with eosin-stained thick fluid on the 7th dpi. These results indicated that B.1.351 and GD108 cause severe inflammation in the upper respiratory tract, with severe inflammation-associated lung injury in macaques; however, the degree of overall lung injury in the B.1.351 group was more severe than that in the GD108 group on the 7th dpi. Indeed, the average lung histopathological score of the B.1.351 group was 0.78 higher than that of the GD108 group (Fig. 5A and B). The above-described results indicate that B.1.351 caused more severe lung and milder trachea pathological changes in macaques than those after GD108 infection.

Moreover, we found that the number of inflammatory cells in the hilar lymph nodes of animals in the GD108 group was reduced compared with that in the B.1.351 group on the 7th dpi (see Fig. S3 at https://figshare.com/s/8204af70a23e2a7da8c8). We also noticed that in B.1.351. group, the hepatic sinus was expanded and filled with eosin-stained thick fluid, and a few inflammatory cells infiltrated into the kidney on the 7th dpi. On the contrary, we did not detect any histopathological changes in the liver and kidney of animals in the GD108 group (see Fig. S4 and S5 at https://figshare.com/s/8204af70a23e2a7da8c8).

### B.1.351 caused a more violent “cytokine storm” in macaques than that after GD108 infection.

**FIG 6:**
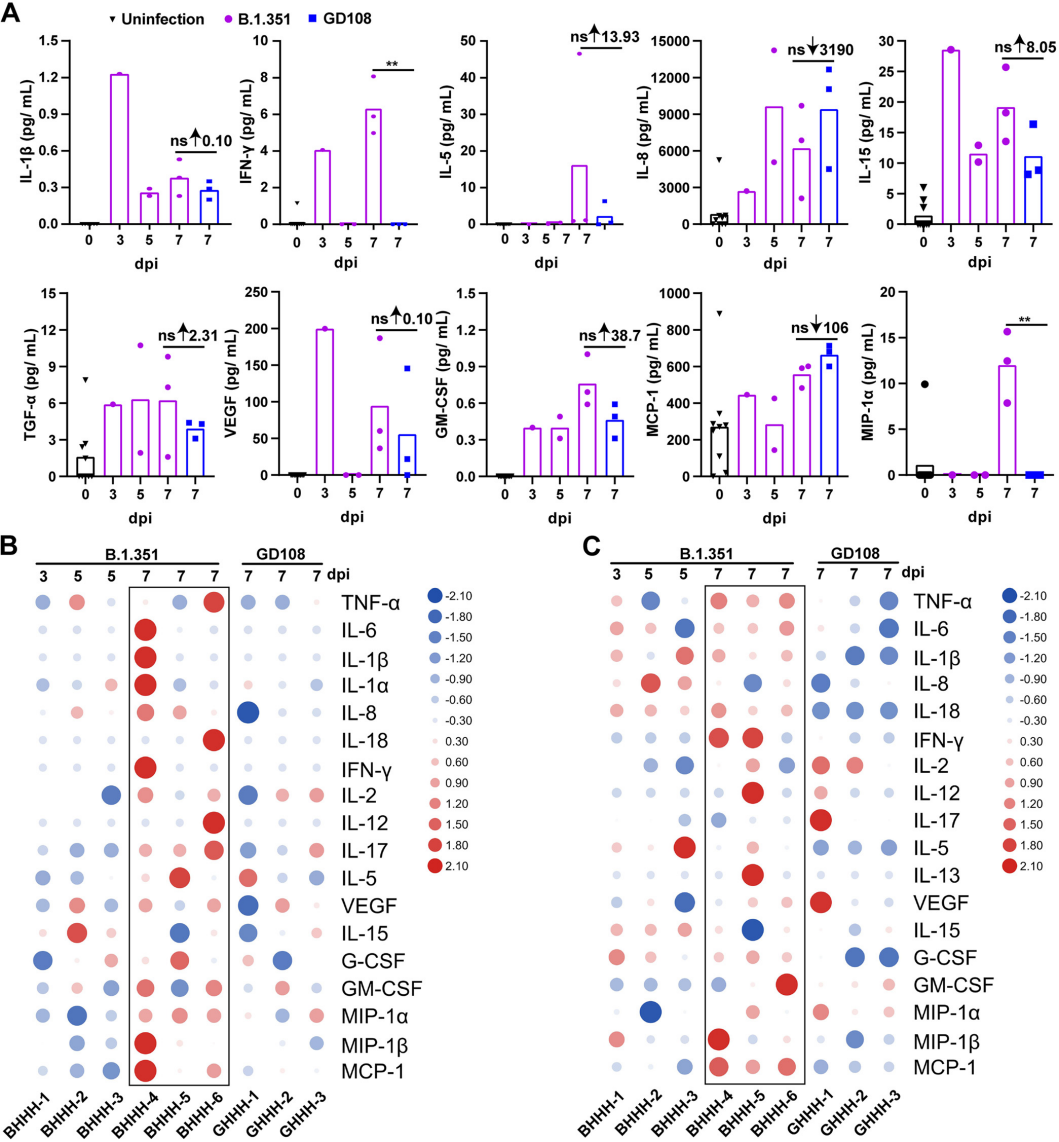
Levels of cytokines and chemokines in macaques induced by infection with B.1.351 and GD108. (A) Serum; (B) BAL fluid; (C) lungs. *n* = 9 at 0 dpi. For B.1.351, *n* = 1 at 3 dpi, *n* = 2 at 5 dpi, and *n* = 3 at 7 dpi. For GD108, *n* = 3 on 7 dpi. ns, nonsignificant; **, *P < *0.01. ↓, decrease; ↑, increase.

To compare the intensity of cytokine storm caused by B.1.351 and GD108, we measured the level of multiple cytokines and chemokines in the serum, BAL fluid, and lung of animals following infection with either B.1.351 or GD108. We detected that the levels of interleukin 1 beta (IL-1β), interferon gamma (IFN-γ), IL-5, IL-8, IL-15, transforming growth factor alpha (TGF-α), vascular endothelial growth factor (VEGF), granulocyte-macrophage colony-stimulating factor (GM-CSF), monocyte chemoattractant protein 1 (MCP-1), and macrophage inflammatory protein 1α (MIP-1α) were dramatically elevated in the serum of macaques postinfection with B.1.351 and GD108, yet levels of IL-1β, IFN-γ, IL-5, IL-15, VEGF, GM-CSF, and MIP-1α in the B.1.351 group were higher than those in the GD108 group on the 7th dpi to various degrees; levels of IFN-γ and MIP-1α in the B.1.351 group especially were markedly higher than those in the GD108 group (*P* < 0.01). On the contrary, only IL-8 and MCP-1 in the B.1.351 group were lower than those in the GD108 group (Fig. 6A). The levels of the IL-4, IL-10, and IL-13 anti-inflammatory cytokines were below the LLOD in both groups (data not shown).

We also found that the levels of multiple proinflammatory cytokines and chemokines such as tumor necrosis factor alpha (TNF-α), IL-6, IL-1β, IL-8, IL-18, IFN-γ, IL-2, IL-12, IL-17, IL-5, granulocyte colony-stimulating factor (G-CSF), GM-CSF, MIP-1α, MIP-1β, and MCP-1 were variously higher in the BAL fluid and lung in the B.1.351 group than those in the GD108 group at 7 dpi (Fig. 6B and C). The levels of IL-4 and IL-10 were also below the LLOD (data not shown). These results indicate that both B.1.351 and GD108 caused a violent cytokine storm in macaques, with B.1.351 causing a more severe effect than that caused by GD108.

### Comparison of cellular immune response in macaques infected with either B.1.351 or GD108.

**FIG 7:**
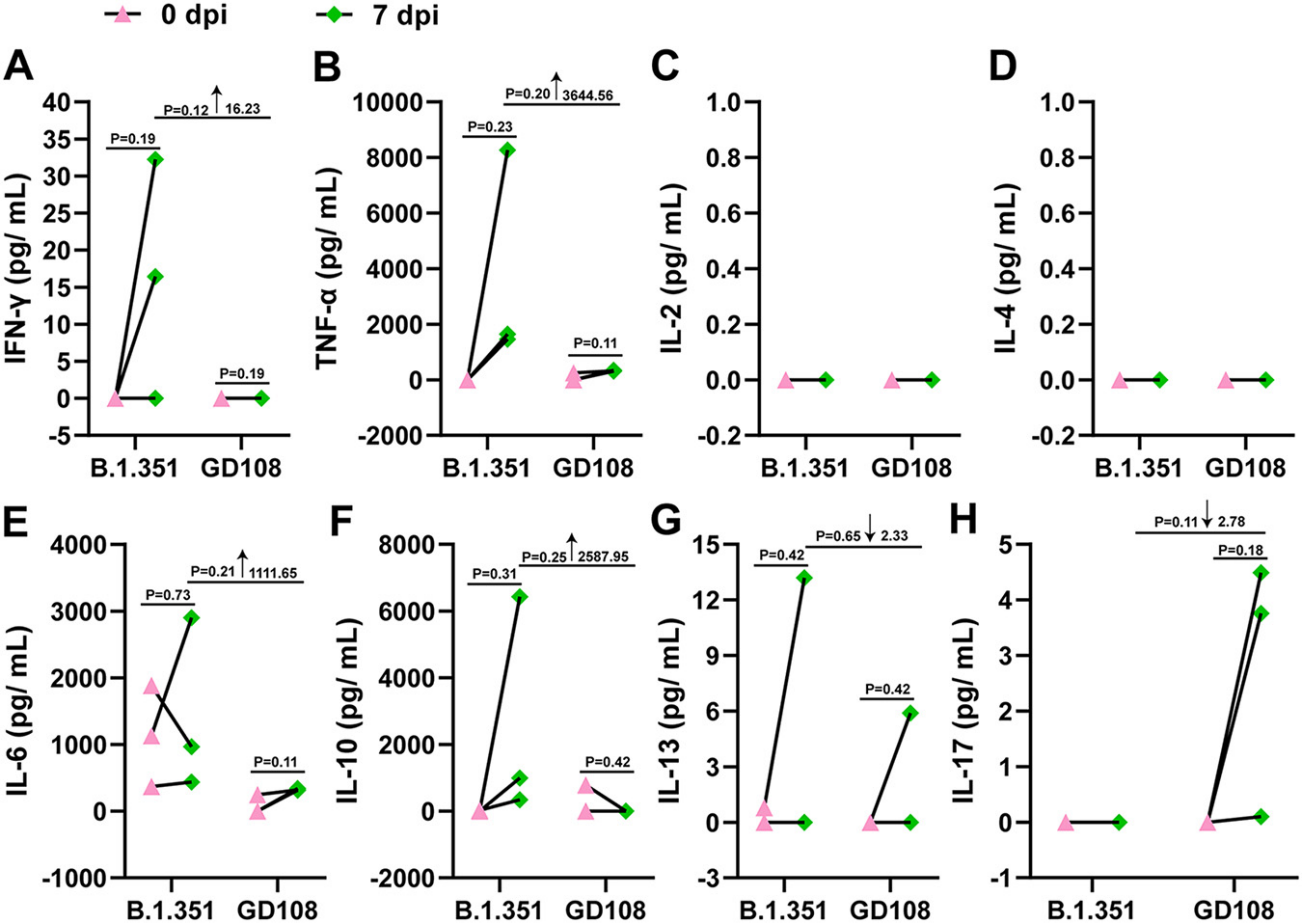
Cytokines secreted by S-specific activated T cells in PBMCs of macaques infected with B.1.351 and GD108 on 0 and 7 dpi. (A) IFN-γ; (B) TNF-α; (C) IL-2; (D) IL-4; (E) IL-6; (F) IL-10; (G) IL-13; (H) IL-17. For B.1.351 and GD108, *n* = 3. ↓, decrease; ↑, increase.

To investigate the cellular immune response caused by B.1.351 and GD108, we collected peripheral blood mononuclear cells (PBMCs) and stimulated them with peptide pools spanning the SARS-CoV-2 S protein. The supernatants were measured for Th1 cytokines IFN-γ, IL-2, and TNF-α; Th2 cytokines IL-4, IL-6, and IL-13; regulatory T-cell (Treg) cytokine IL-10; and Th17 cytokine IL-17 to elevate the spike-specific cellular immune response. Regarding Th1 cytokines, we found that the level of IFN-γ was increased in two out of three macaques, whereas that of TNF-α increased in all three macaques infected with B.1.351; importantly, the levels of both were higher than those in the GD108 group (Fig. 7A and B). We also observed that the levels of IL-2 and IL-4 were below the LLOD in both groups (Fig. 7C and D). Regarding Th2 cytokines, we found that the level of IL-6 was increased in two out of three macaques, whereas that of IL-13 was increased in one out of three macaques infected with B.1.351; again, both levels were higher than those in the GD108 group (Fig. 7E and G). Likewise, the level of IL-10 for Tregs was increased and higher than that in the GD108 group in all three macaques infected with B.1.351 (Fig. 7F). In contrast, the level of IL-17 for Th17 was increased in two out of three macaques infected with GD108, whereas it was not induced by B.1.351 (Fig. 7H). These results indicated that B.1.351 and GD108 induced different types of cellular immune response in macaques.

## DISCUSSION

Assessing the distribution and virulence and investigating the pathogenic characteristics of SARS-CoV-2 variants are currently important aspects of COVID-19 prevention and control. In this study, following the establishment of an NHP model of B.1.351 infection, we compared the differences in the distribution, virulence, and pathogenic characteristics between the B.1.351 and GD108 strains in macaques.

Clinical signs such as decreased body weight, increased body temperature, and activation of the complement system, which manifested in NHPs after their challenge with either B.1.351 or GD108, indicated the successful infection of macaques in both groups. Munster et al. found that SARS-CoV-2 (nCoV-WA1-2020) infection could cause 10% body weight loss and 1.5°C body temperature increase in rhesus macaques. Our clinical results are consistent with Munster’s (14). Our previous study indicated that the level of viral loads in the respiratory system of macaques peaked on day 7 postinfection with GD108 (6, 19). In the present study, we compared the viral loads in swabs and lungs of animals infected with B.1.351 and GD108. We found that the replication capacity of B.1.351 was weaker than that of GD108 in the upper respiratory tract, whereas it was stronger in the lower respiratory tract. This demonstrated a certain difference between the viral replication mode of B.1.351 and GD108. Viral loads in swabs indicated the individual viral shedding, which indirectly reflects the transmission ability during the period of infection. The higher the viral load in respiratory swabs, the longer the shedding time in NHPs after challenge with GD108, which implied that the transmission ability of GD108 was stronger than that of B.1.351 in macaques; this might be one of the reasons the ancestral strain sustained a longer epidemic time than B.1.351 in the real world (16, 20). In contrast, respiratory viruses possessing stronger replication capacity in the lower respiratory tract always tend to be more virulent for human hosts. For example, the low-pathogenic avian influenza virus H9N2 is mainly concentrated in the upper respiratory tract, whereas the highly pathogenic avian influenza viruses H7N9 and H5N1 are mainly concentrated in the lower respiratory tract postinfection (21–23). Therefore, our results suggest that the virulence of B.1.351 might be more severe than that of GD108.

Regarding the pathogenicity of B.1.351, we observed shadows in the lungs of NHPs by chest X ray, as well as severe hemorrhage and slight edema by gross autopsy in macaques infected with B.1.351. Based on histopathological analysis, we found that B.1.351 caused tracheitis and severe inflammation-associated lung injury in macaques. The published study indicated that patients and rhesus macaques could present severe interstitial pneumonia, necrosis, and exfoliation of alveolar epithelium and alveolar structure destruction postinfection with B.1.351 and B.1.1.7 SARS-CoV-2 variants (16). Our lung histopathological changes were similar to those of published studies. Histopathological changes in the thrombus and macrophage phagocytosis of pigment granules indicated the destruction of vascular endothelia in the lungs following infection with B.1.351, in line with the clinical image in humans infected with SARS-CoV-2 (24). In the GD108 group, we also observed tracheitis and inflammation-associated lung injury on the 7th dpi, all of which were consistent with the results in our previous study (6). Numerous studies in animal models and clinical trials indicated that the SARS-CoV-2 prototype could result in pneumonia and lung injury, mainly presenting the inflammatory cells’ infiltration, necrosis, and exfoliation of alveolar epithelium and hemorrhage in lung. The results of this study were similar to published studies (10, 11, 13, 14, 24, 25). However, we verified that the acute lung injury caused by B.1.351 was more severe than that caused by GD108 in macaques on the 7th dpi, which was consistent with the higher level of B.1.351 RNA loads in the lungs. According to published studies, one of the reasons for the poor treatment effect of endotracheal intubation in patients with severe COVID-19 is that the alveoli are filled with a large amount of eosin-stained mucus that impacts the gas exchange in alveoli, which is clearly different from the inflammation-associated lung injuries induced by other viruses, such as the influenza virus (21, 26, 27). In our study, macaques visibly presented the pathological change of eosin-stained thick fluid in the lungs postinfection, which mimicked the typical histopathological change observed in humans infected with SARS-CoV-2. Numerous studies found that NHPs could show the strongest response to SARS-CoV-2 and present the most similar pathogenic characteristics to humans infected with SARS-CoV-2 compared with other animals, suggesting that it might be the suitable model for COVID-19 (3, 6, 28–30). In the present study, these typical pathological changes in the lung suggested that the NHP model is suitable for investigating the pathogenic characteristics of SARS-CoV-2 variants. Previous studies indicated that SARS-CoV-2 could result in liver injury and cytokine-induced hyperinflammation in kidneys (31, 32). In the present study, we also found that the hepatic sinus was expanded and filled with eosin-stained thick fluid, and a few inflammatory cells infiltrated into the kidneys following infection with B.1.351. These histopathological changes were similar to those observed in humans infected with SARS-CoV-2. Combined with histopathology, abnormal blood biochemical indexes, and viral distributions, we suggested that B.1.351 is more likely to cause systemic multiple organ failure. Our results indicate that the pathogenicity of B.1.351 is more severe than that of GD108 in macaques.

The lung is a vital organ for gas exchange, and excessive inflammation in this organ threatens overall body health. As the lung is constantly exposed to harmful pathogens, an immediate and intense defense mechanism, such as inflammation, is required to eliminate pathogens. Therefore, a delicate balance between proinflammation and anti-inflammation responses is essential for lung homeostasis. Following the invasion of SARS-CoV-2 into the host, the body recognizes the virus by pathogen recognition receptors and generates a great deal of proinflammatory cytokines and chemokines to induce inflammation against the invading pathogen. However, no anti-inflammatory cytokines, such as IL-10 and IL-4, are secreted, resulting in an imbalance of proinflammatory and anti-inflammatory responses (27, 33). Therefore, this overload in proinflammatory cytokines and chemokines results in a violent cytokine storm, which is eventually presented as excessive inflammation in the lungs. In the present study, the drastically increased levels of proinflammatory cytokines and chemokines in the serum, BAL fluid, and lung indicated that B.1.351 and GD108 caused a severe cytokine storm in macaques. Some studies have suggested that IL-1β, TNF-α, IL-2, IFN-γ, and IL-6 were markedly upregulated in critical patients after infection with SARS-CoV-2 and thus can be used as biomarkers to determine the severity of disease in patients (27, 31). In our study, the levels of the above-described cytokines and chemokines were higher in the B.1.351 group than those in the GD108 group, indicating that B.1.351 induced a more violent cytokine storm than GD108. This is consistent with the more severe lung histopathological changes caused by B.1.351 in our study. Some research indicated that cytokine levels, such as IL-6, IL-1β, TGF-α, and IL-15, of rhesus macaques were dramatically elevated postinfection with SARS-CoV-2 ancestral B.1, B.1.1.7, and B.1.351. In the present study, B.1.351 and GD108 could also elevate levels of IL-6, IL-1β, TGF-α, IFN-γ, and IL-15 in rhesus macaques (6, 11, 14, 16). Chemokines such as GM-CSF, MCP-1, MIP-1α, and MIP-1β play a critical role in recruiting monocytes and lymphocytes into the inflammatory area (22). In the present study, the levels of the above-described chemokines in the lungs of macaque infected with B.1.351 were higher than those in the GD108 group, indicating that B.1.351 recruited more monocytes and lymphocytes in the lungs than GD108. This finding was also consistent with the lung histopathology in our study. Munster et al. found that levels of MCP-1 and MIP-1α were elevated in rhesus macaques postinfection with SARS-CoV-2. Our results are consistent with Munster’s (14). Previous studies showed that VEGF enhanced vascular permeability, causing organ edema in the body (22). As such, we assumed that the upregulation of VEGF in the serum, BAL fluid, and lung might be one of the reasons for lung edema in macaques postinfection.

Studies have shown the activation of robust T-cell immunity in individuals with asymptomatic or mild COVID-19, with acute-phase SARS-CoV-2-specific T cells displaying a highly activated cytotoxic phenotype that correlated with various clinical markers of disease severity (34). In our study, infection with B.1.351 elevated the levels of IFN-γ, TNF-α, IL-13, and IL-10, whereas infection with GD108 elevated the level of IL-17 in macaques. It is believed that Th1-secreted IFN-γ and TNF-α mainly perform cytotoxic functions, such as resistance to viruses and promoting macrophage activation to induce the inflammatory response; Th2-secreted IL-13 induces B-cell proliferation, and Treg-secreted IL-10 mainly inhibits the excessive immune response, while Th17-secreted IL-17 mainly induces the secretion of multiple proinflammatory cytokines from epithelia and endothelia following viral infection (35). Our results suggest that infection with B.1.351 might mainly activate strong cytotoxic functions and inflammatory response to combat the viral invasion. Meanwhile, the Treg-mediated immunosuppressive function might be initiated to balance the excessive inflammatory response. The previous published study indicated that B.1.351 could increase the level of Th1-secreted IFN-γ in rhesus macaques (19). Sekine et al. found that individuals with asymptomatic or mild COVID-19 present robust T-cell immunity, including Th1 immunity. Our results were similar to the above-described studies (34). Infection with GD108 might mainly induce the Th17 immune response, thus contributing to an increased infiltration of immune cells into the lungs to induce the inflammatory response. A previous study indicated that SARS-CoV-2 prototype polarized Th0 into Th17; however, Th17 could competitively inhibit the polarization of Th0 into Th1 to a certain extent (35), consistent with our results of the infection with GD108. IL-17 was shown to restore T-cell count and reverse lymphopenia, enhance TCR repertoire diversity and the generation of memory CD8^+^ T cells, and improve the trafficking of T cells to the infection site. In the present study, we observed that B.1.351 could not effectively induce the secretion of IL-17. Thus, whether IL-17 can be used to induce the generation and aggregation of T cells to the infection area to fully exert T-cell immunity against B.1.351 and even other variants that could not induce Th17 cellular immunity postinfection remains unclear. Moreover, T cells perform their immune functions mainly through releasing cytokines such as IFN-γ, TNF-α, and IL-17; these excessive levels of T-cell-secreted proinflammatory cytokines could also aggravate the generated cytokine storm postinfection.

There are 11 mutation domains in the S protein of B.1.351, including 3 mutation domains in the receptor binding domain (RBD), 417, 484, and 501 (2). The E484K mutation, which was first found in the Beta variant, has a number of concerning characteristics (16). Several studies have found that the serum-neutralizing ability against E484K-recombinant SARS-CoV-2 in the vaccinated population was decreased compared with that against the prototype strain (19, 36). The affinity between RBD and angiotensin-converting enzyme 2 (ACE2) is one of the most crucial factors for assessing the virus-infecting capacity. In this study, the increased virulence and higher viral spreading ability of B.1.351 might be closely related to the mutation domains in RBD. Numerous published studies that reviewed multiple variants and scanned mutation domains found that the 417, 484, and 501 sites in RBD occur frequently in multiple VOCs, such as Alpha, Beta, Gamma, and even Omicron (BA.2) variants (2, 16, 37–39). The next step will be to use virus rescue techniques to construct viral strains that will be individually mutated at each of these domains to determine which domains are responsible for severe virulence, stronger viral spreading ability, and pathogenic characteristics. In future epidemic prevention and control, we should focus on whether these mutation domains occur in novel SARS-CoV-2 variants.

In summary, the present study successfully established an NHP model of infection with the Beta variant B.1.351. We found that B.1.351 presents increased virulence but reduced viral spreading ability in macaques compared with GD108. Moreover, both B.1.351 and GD108 induce the S-specific T-cell immune response at the early stage of infection, with higher levels of IFN-γ and TNF-α in macaques infected with B.1.351, whereas there were higher levels of IL-17 in those infected with GD108.

## MATERIALS AND METHODS

### Biosafety and ethic statement.

All experiments involving live SARS-COV-2 in this study were performed in an animal biosafety level 4 laboratory (ASBL-4), and all animal studies were conducted in accordance with relevant ethical regulations and approved by the Institutional Animal Care and Use Committee of the Institute of Medical Biology, Chinese Academy of Medical Sciences (Peking Union Medical College, Beijing, China) (approval number DWSP202103004).

### Animal experiments.

In this study, nine Chinese rhesus macaques aged 2 to 3 years were provided and housed at Kunming National Center for Advanced Biosafety Primate Research (Yunnan, China). The nine Chinese rhesus macaques were divided into two groups, the SARS-CoV-2 Beta variant B.1.351 (B.1.351) group (six Chinese rhesus macaques, abbreviated BHHH) and the GD108 strain (GD108) group (three Chinese rhesus macaques, abbreviated GHHH). We used the GD108 ancestral strain of SARS-CoV-2 as the comparison for the B.1.351 SARS-CoV-2 Beta variant in our study. In the B.1.351 group, six HHHs were infected with 1 × 10^6^ 50% tissue culture infective dose (TCID_50_) of B.1.351 via intratracheal and intranasal routes (1:1). To conform to the principles of the 3Rs (replacement, refinement, and reduction), the macaques HHH-4, HHH-5, and HHH-6 in the B.1.351 group were shared in another study conducted by our lab as a challenge control group (19). Similarly, in the GD108 group, three HHHs were infected with 1 × 10^6^ TCID_50_ of GD108 via intratracheal and intranasal routes (1:1). Our previous published studies showed that macaques infected with GD108 present typical symptoms and progressive exacerbation from 0 to 7 days postinfection (dpi). Both levels of viral loads and cytokines reach their peak with macaques presenting severe lung injury on 7 dpi (6, 19, 40). In this study, we used the same virus strain, GD108; rhesus macaques of the same species, age, and origin; the same laboratory; and the same experimenters as our previous published studies. Therefore, body weight and temperature were measured at 0, 1, 3, 5, and 7 dpi, and the viral distribution, virulence, and pathogenic characteristics of B.1.351 and GD108 groups were adequately compared at 7 dpi to conform to the principles of the 3Rs. In the B.1.351 group, 1, 2, and 3 macaques were sacrificed at 3, 5, and 7 dpi, respectively. In the GD108 group, 3 macaques were sacrificed at 7 dpi. The detailed animal experimental process is shown in Fig. 1A.

### Chest radiography.

According to the method established in our previous study, chest X-ray imaging at 55 to 75 V and 8 to 12.5 mA was performed on the indicated days using a MobileCooper mobile digital medical X-ray photography system (MobileSparkler; Browiner, China) (6). Images were independently evaluated by experienced and qualified radiologists using a 4-pattern approach (analyses of consolidation, interstitial areas, nodules or masses, and atelectasis).

### Hematological examination.

Blood was collected from each animal at each time point. Subsequently, hematological examination was performed using an automatic blood analyzer.

### qRT-PCR.

Trachea, bronchus, hilar lymph node, lung, and other organ samples were obtained after weighing, homogenized, and centrifuged (3,000 rpm for 10 min) to obtain viral supernatants. Bronchoalveolar lavage fluid (BAL fluid) was centrifuged (3,000 rpm for 10 min) to obtain viral supernatants. Consequently, viral RNA was extracted using the magnetic viral nucleic acid kit (catalog no. DP614; Tiangen, China) according to the manufacturer’s instructions. The viral genomic RNA (gRNA) and viral subgenomic RNA (sgRNA) that represent the replicating virus were quantified in each sample by using qRT-PCR. Viral loads in each specimen were determined according to the standard curve. Specimens with a viral load of less than 1 copy/μL could not be accurately measured using this assay. Thus, in this assay, the lower limit of detection (LLOD) of viral RNA was set to 1 copy/μL. The LLOD of lung and other organs were 2,500 copies/g, whereas the LLOD of BAL fluid and swabs was 500 copies/mL.

### H&E staining.

Lungs and other organs were collected and fixed in 4% paraformaldehyde (catalog no. P1110; Solarbio, China), embedded in paraffin, sectioned, and stained for histopathological analysis using H&E staining. Sections were evaluated by experienced and qualified pathologists in a double-blinded manner. According to the published research and our previous studies, lung pathological scores on all six-part lung lobes of each macaque were performed, and then, the mean of six-part lung lobes was taken to represent the pathological score of that macaque. Inflammation, hemorrhage, thickening, and necrosis in the alveoli and pulmonary interstitial tissues were scored on a 0- to 4-point scale as follows: no injury, score of 0; injury in 0 to 25% of the field, score of 1; injury in 25% to 50% of the field, score of 2; injury in 50% to 75% of the field, score of 3; and injury in 75% to 100% of the field, score of 4 (6, 11, 14, 19).

### Multiple-cytokine profiling.

Lungs, bronchoalveolar lavage fluid (BAL fluid), and blood were collected from each animal at each time point. Lung samples were weighed, and homogenate supernatants were prepared for analyses. Blood was centrifuged (3,000 rpm for 10 min), and the serum was collected for analyses. For specific T-cell response evaluation, freshly isolated peripheral blood mononuclear cells (PBMCs) were stimulated with peptide pool spanning the SARS-CoV-2 spike (S) protein for 20 h at 3 × 10^5^ cells per well. The concentration of each peptide was 5 μg/mL. The peptide pool was generated as follows: a panel of consecutive 15-mer peptides with overlapping 9 amino acids were synthesized to encompass the entire S protein and mixed as a single peptide pool. After stimulation, the supernatants were collected. All samples were assayed using the Millipore Milliplex nonhuman primate cytokine magnetic bead panel premixed immunology multiplex assay, and the concentration of each sample was calculated using a standard curve. The concentrations of cytokines in lungs and BAL fluid were translated into log_2_ values and then calculated using the Z-score to perform comparisons between the two groups.

### Statistical analysis.

All statistical analyses were performed using GraphPad Prism 8.0 (GraphPad Software, Inc.). Virus loads were transformed into log_10_ values to analyze. Statistical significance was evaluated between the two groups by using Student’s test. *P* values of <0.05 indicate a difference.

### Data availability.

All data generated in this study are available from the corresponding author upon reasonable request.
